# Age Limit and Radiotherapy Option for Sarcomatoid Carcinoma of the Larynx: A Case Report with Literature Review

**DOI:** 10.7759/cureus.3023

**Published:** 2018-07-22

**Authors:** Mina Fransawy Alkomos, Michael Rizk, Goubran Eskander, Ahmed Elkheshen, Rupak Mahendhar, Amir Shahbaz, Paria Zarghamravanbakhsh, Phoebe Younan, Nasim Golchin, Issac Sachmechi

**Affiliations:** 1 Research, California Institute of Behavioral Neurosciences & Psychology, Sacramento, USA; 2 Otolaryngology, Ain Shams University, Cairo, EGY; 3 Internal Medicine, The Icahn School of Medicine at Mount Sinai, New York, USA; 4 Internal Medicine, Icahn School of Medicine, Mount Sinai/Queens Hospital Center, New York, USA; 5 Internal Medicine, Icahn School of Medicine at Mount Sinai/Queen Hospital Center, New York, USA; 6 Endocrinology, Icahn School of Medicine at Mount Sinai Queen Hospital Center, New York, USA; 7 Faculty of Medicine, Ain Shams University, Cairo, EGY; 8 Endocrinology, Icahn School of Medicine at Mount Sinai/Queens Hospital Center, Queens, USA; 9 Internal Medicine, Icahn School of Medicine at Mount Sinai/Queens Hospital Center, New York, USA

**Keywords:** carcinosarcoma, spindle cell carcinomas, laryngeal sarcomatoid carcinoma

## Abstract

Sarcomatoid carcinomas, also known as spindle cell carcinomas (SPCCs), are rare carcinomas, predominantly developing in the lung. They have lots of features of sarcoma in their histological features. The standard laryngeal carcinoma classification is based on tumor size, lymph node affection, and metastasis (TNM), it is the classification scheme of the American Joint Committee on Cancer Staging (AJCC), and it is used in the same way for stage spindle cell carcinoma (SPCC). We present a case report of a young female along with a literature review of sarcomatoid carcinoma of the larynx.

## Introduction

Sarcomatoid carcinomas, also known as spindle cell carcinomas (SPCC), are rare carcinomas that predominantly develop in the lung. They have lots of features of sarcomas in their histological features [1-3]. In the larynx, sarcomatoid carcinomas are considered high-grade variants of squamous cell carcinomas, with no randomized clinical trials, conducted to specify a treatment modality [2]. The standard laryngeal carcinoma classification scheme of the American Joint Committee on Cancer Staging (AJCC) is used in the same way as to stage SPCC [3]. The classification is based on tumor size, lymph node affection, and metastasis (TNM) [3]. Most laryngeal SPCCs give symptoms early, predominantly hoarseness of voice. The reason for hoarseness of voice is that they most commonly appear as glottic pedunculated polypoidal masses (T1 or T2 lesions) with minimal invasion of the underlying stroma. The presenting gross appearance allows early wide complete transoral local excisions [3]. Scholars suggested good disease-controlled outcomes for early-stage glottic sarcomatoid carcinomas when treated with irradiation, comparing favorably with early glottic squamous cell carcinomas [4].

## Case presentation

**Figure 1:**
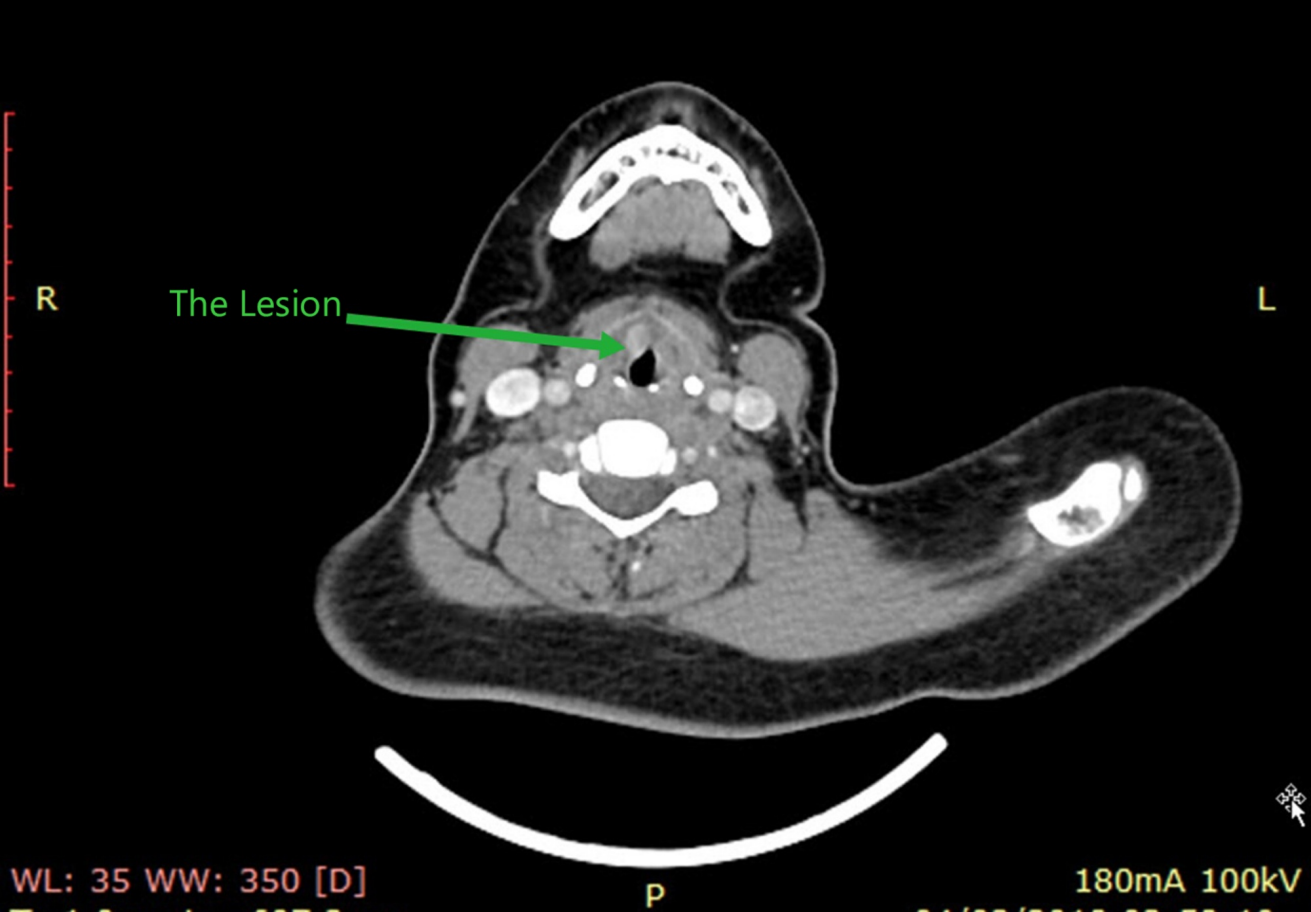
CT scan findings: A lesion at the glottic level, without any lymphadenopathy. CT: computed tomography

A 24-year-old female patient presented to the otolaryngology clinic with a six-month history of progressive hoarseness of voice. In addition, she has a recent history of mild dyspnea on exertion and dry cough. The patient did not have any weight loss nor dysphagia. The patient was a cigarette smoker of around one pack per day for five years. There was no past family history of cancer, and she did not have any medical illness of significance. The patient also did not have any prior history of radiotherapy. In the clinic, fiberoptic nasoendoscopy showed a right vocal fold mass reaching the anterior commissure. The vocal fold mobility was normal. The neck examination was unremarkable. Our clinical impression, at this stage, was that the patient had early glottic laryngeal cancer. Consequently, the patient had a computed tomography (CT) scan showing the mass with no cervical lymphadenopathy (Figure 1).

**Figure 2:**
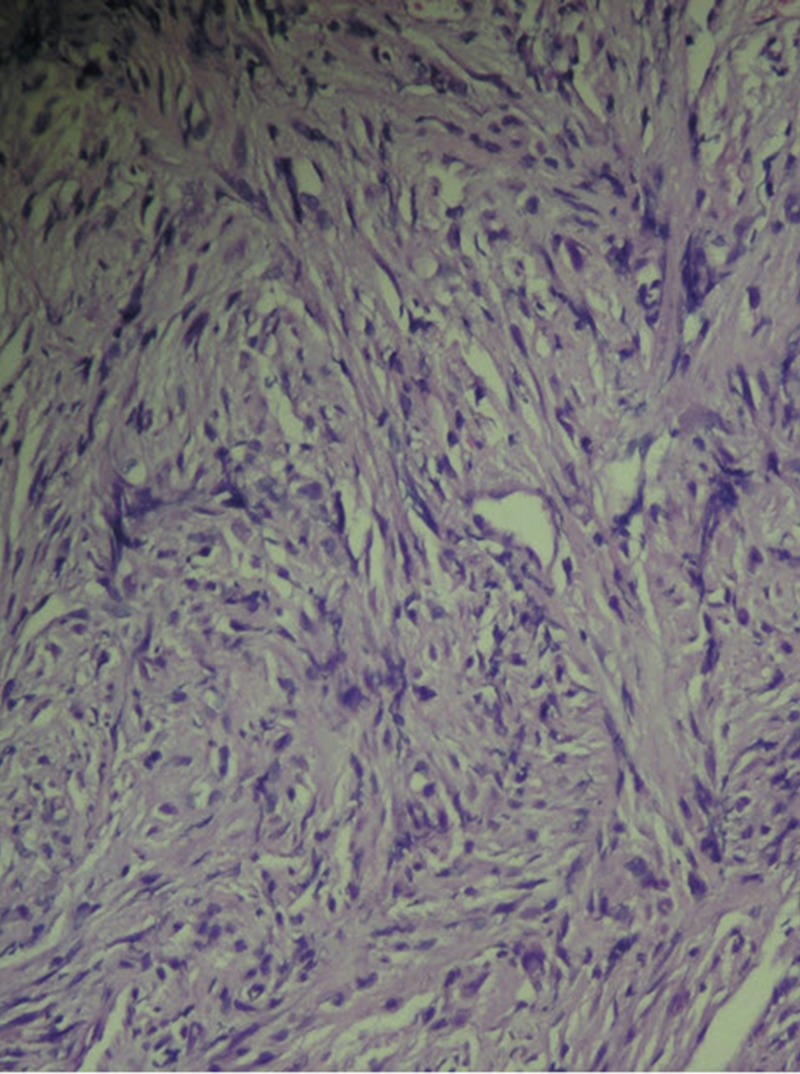
Histopathology stained with H&E: Spindle cells with short fascicles and pleomorphic nuclei.

**Figure 3:**
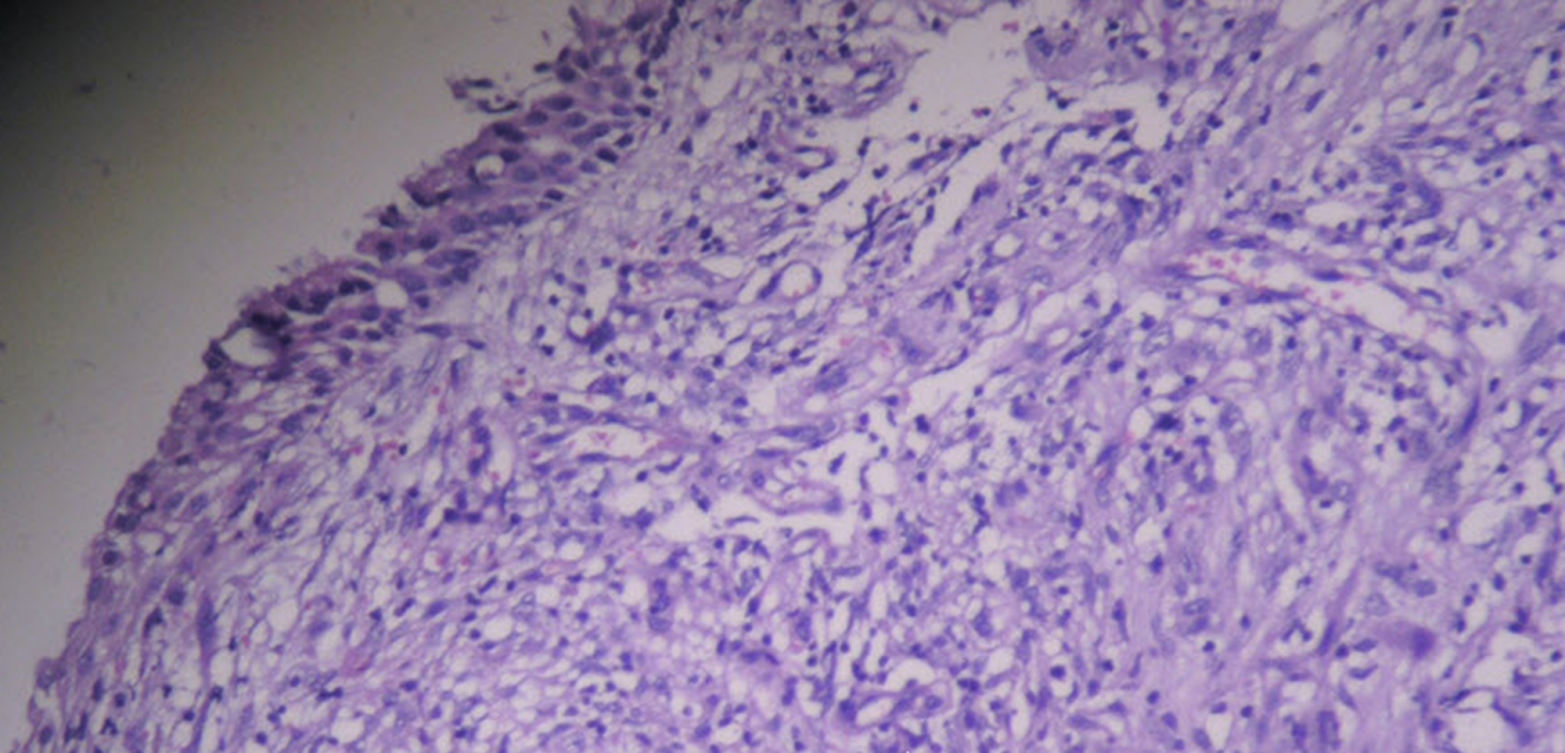
Histopathology: Cells eroding mildly dysplastic epithelium.

**Figure 4:**
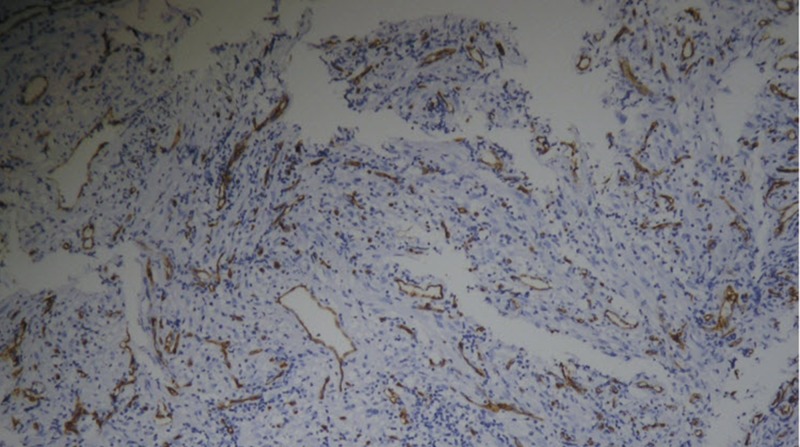
Histopathology: CD34 positive internal control and negative in tumor cells.

The vocal fold mobility was normal. The neck examination was unremarkable. The patient underwent a laryngotracheoscopy under general anesthetic, with a biopsy taken from the lesion, which appeared to be arising from the right vocal fold without a subglottic extension. The initial histopathology report confirmed sarcomatoid carcinoma and subsequent immunohistochemistry was positive for epithelial membrane antigens (EMA), cytokeratin CK 5/6, and cytokeratin AE1/AE3AE 1/3 (Figures 2-4).

**Figure 5:**
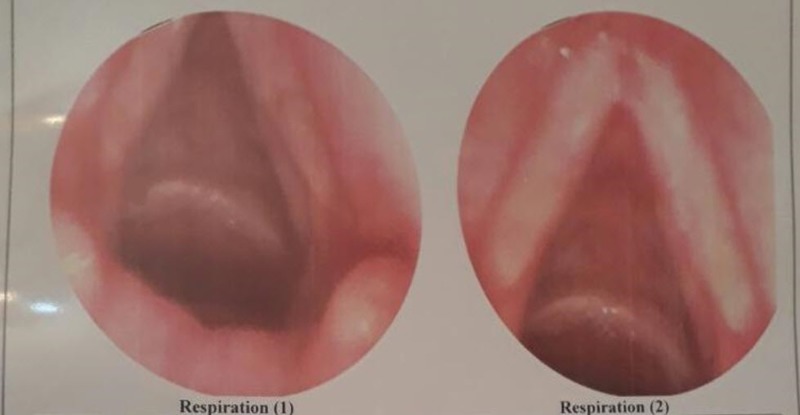
Follow-up by laryngoscope after six months showed no recurrence. Respiration (1) shows the posterior commissure and respiration (2) shows the anterior commissure.

The patient’s spindle cell (sarcomatoid) carcinoma stage was T2N0M0 according to the AJCC cancer staging system for laryngeal carcinomas. The options for management were evaluated by the head and neck multidisciplinary team, who preferred a transoral surgical excision as a modality of treatment. We discussed the treatment options with the patient, who refused surgical intervention. Consequently, she received intensity-modulated radiotherapy (IMRT). The patient had followed up at a six-month interval, and she remains free of the disease (Figure 5).

## Discussion

Methods

We also conducted a review of the literature based on a search performed on June 9, 2018, in PubMed. We discovered 167 articles, and on further screening, we excluded 103 articles, as they were not related to our topic or we could not download it. Then, 49 studies were excluded in the full-text analysis due to irrelevant disease. Fifteen articles were included in our review of the literature. We reviewed 59 patients with laryngeal carcinosarcoma having different ages, sexes, and modalities of carcinosarcoma treatment. The following table illustrates the review of the included patients (Table 1).

**Table 1:** Clinical characteristics of patients with laryngeal sarcomatoid carcinoma described in the literature (n=59) M = Male, F= Female, Mo = month Rt = right, Lt = left, FOD: free of disease, VC = vocal cord, SCC = squamous cell carcinoma, SPCC = spindle cell carcinoma, Y = year, GCT = Giant cell tumor, EMA = Epithelial membrane antigen, Ds = Disease, Rad. = Radiotherapy, TL = Total laryngectomy, RND = Radical neck dissection, SSL = Subtotal supraglottic laryngectomy, SCPL-CHP = Suprecricoid partial laryngectomy. pseudosarcoma, indicating the view that the spindle-shaped cells are reactive rather than neoplastic.

ID	Article	Age (Years) /Gender	Specific Habits/ Symptoms/Duration	Site/Classification		Treatment	Pathology Report of Surgical Specimen	Subsequent course/Follow-Up
1	Randall et al. [5]	58/M	Smoker/hoarseness for two Mo	Rt anterior commissure/T1bN0M0		Local excision	Pseudo-Sarcocarcinoma in situ	Neck metastasis four Mo, and FOD eight Y later
		56/M	Smoker/hoarseness for two Wks	Lt anterior commissure/ T2N1M0		SSL	Carcinoma in situ and invasive epidermoid carcinoma pseudo sarcoma	Neck metastasis nine Mo, lt RND, one node has cancer/FOD four M0 before death from MI
		43/F	Smoker/hoarseness for four Mo	Lt TVC/T1aN0M0		Lt HL	Invasive epidermoid carcinoma with underlying pseudosarcoma	FOD nine and a half Y
		74/M	Smoker/hoarseness for one Y	Anterior commissure/T2N0M0		Frontal HL	Pseudosarcoma	FOD four Y
		60/M	Smoker/hoarseness for three Mo	Rt FVC/T2N0M0		SSL with foldover Rt RND	Osteosarcoma	FOD three and a half Y
		67/M	Smoker/hoarseness for one Mo	FVC/TV bilateral/T3N1M0		TL	Pseudosarcoma	FOD 12 Mo
		55/M	Dysphagia for three Mo	Epiglottis rt T3N1M0		SSL, Bilateral RND	Pseudosarcoma	Died with cancer three Y
		80/F	Dysphagia for eight Mo	Lt AE/ T3N0M0		SSL, LT RND	Pseudosarcoma, carcinoma in situ	Died Free of Ds two Y
		80/M	Dysphagia for three Y	Lt TVC/ T3N0M0		TL	Pseudosarcoma	Died with cancer 13 M0
2	Appelman et al. [2]	62/M	Smoker/hoarseness for one Y/dyspnea for one wk	Rt ventricle, Rt VC		Radiotherapy	Fusion of SCCt and SPCC	Local recurrence, 10 Mo/died one and a half Y
		51/M	Smoker/hoarseness for three M/otalgia	Anterior half of Lt VC		HL	SCC with sarcoma-like areas	FOD 15 Y
		72/M	Hoarseness for three mo	Base of the epiglottis		Rd	Fusion of SCC and spindle cells	Local recurrence, Five Mo, radium implant/died 14 months later
		49/M	None	Right arytenoid		Snare excision +radiotherapy	SCC intermixed with spindle cells; osteoid and chondroid areas	Cervical node metastasis, one Mo/died two and a half Y
		56/M	Hoarseness for three mo	Left true cord		None	Predominant bizarre spindle cell neoplasm	Died five mo
		59/M	Difficulty swallowing for six mo	Hypopharynx, false cord		None	SCC intermixed with spindle cells; osteoid and chondroid areas	Died eight Mo
		48/M	Dysphagia for seven Mo	Right, true cord		Rd then local recurrence, Four yr.; total laryngectomy	SCC intermixed with spindle cells; osteoid metaplasia	Died, five and a half Y, cervical node metastasis
		42/M	Hoarseness (nine mo) neck pain (one Mo)	Right, true cord		HL	Fusion of SCC and spindle cells	Alive and ell after 13.5 Y
		59/M	Hoarseness for two yr, dysphagia for three Mo.	Both true cords and commissure		TL/Metastasis in cervical lymph nodes, three Mo then RD	Fusion of SCC and spindle cells	Died, 10 Mo
		73/F	Hoarseness, dysphagia, weight loss, six Wks	Right ventricular fold		TL/regional recurrence, cervical lymph node metastasis, 10 Mo followed by radiotherapy	Fusion of SCC and spindle cells	Died two and a half Y later
		77/M	Hoarseness for 10 mo	Right, true cord		TL	SCC with demarcated sarcoma-like areas	FOD one and a half year later
3	Katholm et al. [6]	50/M	Smoker/hoarseness	LT VC/T1AN0M0		Radiotherapy	Interlacing bundles of large spindle-shaped cells with pleomorphic nuclei and nucleoli.	FOD 22 Mo
4	Alguacil-Garcia et al. [7]	59/M	Smoker/hoarseness for one year	Rt VC		TL	Sarcomatoid carcinoma	FOD two Y
5	Hellquist et al. [8]	64/M	Hoarseness of voice for seven Mo	VC		TL	SPCC	
		66/F	Hoarseness of voice for eight Mo	Epiglottis		Rad	SPCC	FOD three and a half Y
		54/M	Hoarseness of voice for three Mo	VC		TL	SPCC	FOD three Y
		69/M	Hoarseness of voice for four Mo	VC		Excision, rad	SPCC	FOD one and a half Y
		71/M	Hoarseness of voice for two Mo	VC		Rad/then recurrence after five Y, then laryngofissure	SPCC	FOD eight and a half Y
		62/F	Hoarseness of voice for three Mo	VC		Excision	SPCC	FOD one and a half Y
		57/M	Hoarseness of voice for four Mo	VC		TL+Radio	SPCC	FOD one and a half Y
		36/M	Hoarseness of voice for four Mo	Subglottis		Excision + Radio	SPCC	FOD three Y
		63/M	Hoarseness of voice for five Mo	VC		Radio	SPCC	FOD two Y
		75/F	Hoarseness of voice for six Mo	VC		Radio	SPCC	LNs metastasis After one Y died with the ds after three Y
		59/M	Hoarseness of voice for three and a half Mo	VC		Radio	SPCC	FOD 11.5 Y
		67/M	Hoarseness of voice for two and a half Mo	VC		Excision+radio	SPCC	FOD six Y
		57/M	Hoarseness of voice for two Mo	VC		Excision	SPCC	FOD 10.5 Y
		69/M	Hoarseness of voice for two Mo	VC		Excision	SPCC	FOD five Y
6	Lassaletta et al. [9]	48/F	Smoke 40 Cig/day/ hoarseness and intermitted stridor	Lt VC		Functional neck dissection and total laryngectomy	GCT Lt VC/SPCC in subglottic/positive for EMA, cytokeratin CK 5/6 and cytokeratin AE1/AE3AE 1/3, keratin 8, anion exchange keratin 1,3. Negative for S100	FOD six Mo
7	Miyahara et al. [10]	88/M	Smoker/hoarseness	RT VC/T2N0		TL	Carcinosarcoma	Died seven Y later of old age not the Ds
		86/M	Smoker/hoarseness	RT VC/ T1aN0		Total laryngectomy	Spindle cell carcinoma	FOD six Y
		68/M	Smoker/hoarseness	Anterior commissure/T1bN0		Rad.	Spindle cell carcinoma	FOD six Y
		76/M	Smoker/hoarseness	VC/T1aN0		Extirpation	Spindle cell carcinoma	FOD six Y
8	Onishi et al. [11]	73/M	Smoker/hoarseness two Mo	Lt VC/T1aN0M0		Partially resection/radio	invasive poorly differentiated SCC with massive sarcomatoid changes (spindle cells)	Died eight M0 after diagnosis with the tumor
9	Franzen et al. [12]	61/M	Hoarseness four Mo, dysphonia two Wks	Right VC and anterior commissure/T2 N0 M0 R0 G3		Partial laryngectomy and modified radical neck dissection		
		53/M	Smoker/swallowing difficulty 2 Mo/ wt loss > 12 kg	Right laryngeal wall of the sinus piriformis and the aryepiglottic fold/T3 N2B Mo		Right-sided laryngeal hypopharyngeal resection and neck dissection	Light microscopic and immunohistochemically becomes a biphasic tumor(carcinosarcoma)	
10	Boamah et al. [13]	67/M	Smoker/hoarseness 2 Mo, dysphagia	Anterior commissure/T1		Excision + radiotherapy	Spindle cell carcinoma, positive for EMA, CK 5/6 and AE 1/3, and a high MIB-1 but negative for myoD1, SMA, desmin, and myf4	Improved
11	Rutt et al. [14]	69/M	Smoker (15 Y) quit (30 Y prior)/ Dysphonia/six Mo	Rt VC/ T1N0M0		Micro flap excision then wider excision	SCC, spindle cell variant	FOD one Y
		61/M	Rough, unstable voice with phonatory breaks	Lt VC/T1N0M0		Surgical excision	SCC, spindle-cell variant	Recurrence six Mo, Co2 laser excision with margin control/ free, six Mo
12	Zhang et al. [15]	66 /M	Smoker/hoarseness	Bilat vocal folds/ T3N0M0		TL	SCC, malignant fibrous histiocytoma	88 Mo/AWD
		64 /M	Smoker/ hoarseness	Lt false VC, laryngeal Ventricle/ T2N0M0		Vertical partial laryngectomy	SCC, malignant fibrous histiocytoma	62/AWD
		59 /M	Smoker/hoarseness	Lt false VC, laryngeal ventricle/T2N1M0		SCPL-CHP + RND	Poorly differentiated Ca, leiomyosarcoma	60/DOD
		60 /M	Smoker/hoarseness, dyspnea	Lt false vocal fold, bilat vocal folds/T3N0M0		Total laryngectomy + RND	Mucinous adenocarcinoma, fibrosarcoma	24/AWD
		57 /M	Smoker/hoarseness, dyspnea	Bilat vocal folds/T3N0M0		Total laryngectomy + SND	Poorly differentiated Ca, osteosarcoma	20 Mo/AWD
		50 /M	Abnormal throat sensation	Epiglottis, R aryepiglottic fold/T3N1M0		Total laryngectomy + RND	Poorly differentiated Ca, embryonal rhabdomyosarcoma	17 Mo/AWD
		42 /M	Smoker/hoarseness	R VC/T2N0M0		Vertical partial laryngectomy	Ca in situ, leiomyosarcoma	13 Mo/AWD
13	Zheng et al. [16]	55/M	Smoker/neck mass	Lt pyriform sinus		Excision, reconstruction, and neck dissection	SPCC	Free eight M0
		62/M	Smoker/hoarseness	Lt VC		TL+ dissection	SPCC	Pulmonary metastasis six M0
		57/M	Smoker/foreign body	Posterior wall of hypopharynx		Total hypopharyngectomy	SPCC	Free five and a half M0
14	Bostanci et al. [17]	60/M	Smoker/hoarseness five Y	Rt VC/T1N0M0		Excision + Radiotherapy	Atypical spindle cells/epithelial component positive cytokeratin and p63	FOD 12 M0
15	Rao et al. [18]	45/M	Smoker 10Y / hoarseness, difficulty in swallowing and breathing six Mo	Lt VC/ T1N0M0		Mass excision	Pleomorphic spindle-shaped cells	FOD three Mo

Results

The included articles described a total of 59 patients, 52 of which were males and seven were females. The age range was between 36 and 88 years. The reported cases were from 1960 till 2016. Most of the patients were smokers, and most of them underwent total laryngectomy followed by radiotherapy. On the other hand, eight patients received radiotherapy only, while the rest had different types of surgery. The general survival outcome was better with a combination of both surgery and radiation, compared to radiotherapy alone.

Discussion

Carcinosarcoma is a rare lesion; it can be present in many body parts, including the larynx. The larynx appears to be an unusual site, as only a few cases can be found in the literature. Carcinosarcoma is considered a malignant tumor composed of both epithelial and mesenchymal components. It accounts for less than 1% of all malignant tumors of the larynx and hypopharynx [19]. In our review of the literature, we noticed that there is a broad age range for laryngeal carcinosarcoma (36-88 years), while our case was 24 years old. Despite being a rare condition, spindle cell carcinoma should be considered a valid diagnosis in any age group, especially if the patient presents with hoarseness of voice or dyspnea and a history of smoking. Surgical intervention was the first line of management in the treatment of most of the laryngeal carcinosarcomas mentioned in our literature with a favored outcome. However, scholars suggested good disease-controlled results for early-stage glottic sarcomatoid carcinomas when treated with irradiation, comparing favorably with early glottic squamous cell carcinomas [4]. We had eight patients in our review that received radiotherapy only, without any surgical intervention [2,6,8,10}. These patients had different ages, cancer stages, and laryngeal cancer locations. Five out of the eight patients who were treated only with radiation have no evidence of the disease at a range of two to 11.5 years while the other three developed a recurrence and died of the disease later on. So radiotherapy might be a valid alternative option instead of surgical therapy in selected patients. Our patient has stage T2N0M0 glottic carcinoma. Due to the early detection of the tumor and its site, the surgical excision was a valid and reasonable option for management. However, the patient preferred the radiotherapy option. The follow-up plan will be the same as for other glottic carcinomas.

## Conclusions

Spindle cell carcinoma (SPCC) or sarcomatoid carcinoma of the larynx is a rare, highly malignant variant of squamous cell carcinoma. It can present at an early age with symptoms and diagnosis is possible in the early stages. Although in some patients, surgery is the best modality of therapy, radiation only can be a reasonable alternative.
